# Hepatic arterial administration of ginsenoside Rg3 and transcatheter arterial embolization for the treatment of VX2 liver carcinomas

**DOI:** 10.3892/etm.2012.873

**Published:** 2012-12-21

**Authors:** YANG YU, CHUNLE ZHANG, LINGJUN LIU, XIAO LI

**Affiliations:** 1 Departments of Gastroenterology and Hepatology, West China Hospital, Sichuan University, Chengdu 610041, P.R. China; 2 Department of Radiology, West China Hospital, Sichuan University, Chengdu 610041, P.R. China

**Keywords:** ginsenoside Rg3, VX2 liver tumor, transcatheter arterial embolization, angiogenesis, apoptosis

## Abstract

Ginsenoside Rg3 has been demonstrated to inhibit tumor cell proliferation and angiogenesis. However, its effect on liver tumors when administered via the hepatic artery has not been investigated. The purpose of this study was to evaluate the therapeutic effect of hepatic artery administration of Rg3 combined with transcatheter arterial embolization (TAE) in the treatment of liver tumors. A total of 48 rabbits with VX2 liver tumors were randomly divided into four groups: Group 1, Rg3; Group 2, TAE; Group 3, Rg3 and TAE; and Group 4, control. Abdominal contrast computed tomography (CT) scans were performed 2 weeks before and after intervention to assess tumor growth. Immunohistochemical staining was used to detect the expression of the angiogenesis biomarkers CD31 and VEGF, and the cell apoptosis marker caspase-3. Semi-quantitative RT-PCR and western blotting were employed to detect the expression of the caspase-3, Bax and Bcl-2 apoptosis-related genes and proteins. In addition, HepG2 cells were treated with Rg3 at different concentrations (0, 25, 50, 75 and 100 mg/l) *in vitro*. An MTT assay and western blot analysis were used to analyze the cell proliferation and VEGF expression. Compared with the other experimental groups, the Rg3 and TAE group expressed significantly lower levels of CD31 and VEGF (P<0.05), significantly increased levels of the pro-apoptotic genes caspase-3 and Bax (P<0.05), and significantly reduced levels of anti-apoptotic Bcl-2 at the mRNA and protein levels (P<0.05). *In vitro*, Rg3 inhibited HepG2 cell proliferation and downregulated VEGF expression significantly. These results indicated that ginsenoside Rg3 combined with TAE may effectively inhibit tumor growth by inhibiting tumor angiogenesis and inducing cancer cell apoptosis.

## Introduction

Transcatheter arterial embolization (TAE) for the treatment of liver cancers is a minimally invasive procedure which may improve survival and quality of life (QOL) (1,2). However, the currently used embolic agents do not congest microvessels sufficiently and therefore TAE is a palliative treatment rather than a curative therapy (3). In addition, as a result of cancer cell ischemia and hypoxia, tumor angiogenesis may be induced by TAE and may contribute to tumor recurrence and metastasis (4,5). Therefore, liver tumors almost always require repeated TAE.

Ginseng, a highly valued herb from East Asia has been used by the Chinese for thousands of years. There is extensive evidence on the beneficial effects of ginseng (6). The major biologically active components of ginseng are various types of ginsenosides (7). Of these, ginsenoside Rg3 has been shown to inhibit cancer cell proliferation (8) and angiogenesis (9). The route by which a drug is delivered may have a significant effect on its therapeutic effect. To date, the therapeutic effect of Rg3 on liver tumors when administered via the hepatic artery has not been investigated. The liver VX2 carcinoma derived from a Shope virus-induced papilloma is an accepted and suitable model for interventional therapy due to its continuous tumor growth and rich arterial supply (10). Using rabbit liver VX2 carcinoma models, we evaluated the therapeutic effect of hepatic artery administration of Rg3 combined with TAE for the treatment of liver tumors.

## Materials and methods

### Sample and randomization

This study was approved by the Institutional Animal Ethical Committee of Sichuan University (Chengdu, China). A total of 50 adult male New Zealand white rabbits weighing 2.3–4.2 kg were acquired from the Experimental Animal Center of West China Medical Center, Sichuan University. The VX2 allografts were provided by The Union Hospital, Huazhong University of Science and Technology, Wuhan, China. The tumors were implanted in lateral muscles of the hind limbs of donor rabbits. The tumor tissue required for implantation in liver was obtained 2 weeks later. After ketamine hydrochloride (40 mg/kg; ShuangHe Pharmaceutical Co., Ltd., Beijing, China) and xylazine (5 mg/kg; Yaji Pharmaceutical, Shanghai, China) anesthesia was intramuscularly injected, laparotomy was performed. A section of tumor tissue (2×2×2 mm) was implanted into the left lobe of the recipient liver. The abdominal wall was closed layer by layer. CT scans of abdomen were performed on all animals 2 weeks after tumor tissue implantation. Animals bearing a tumor >10 mm in diameter were included in the experiment. Rg3 was extracted from a sample of ginseng from Northeast China provided by Yatai Pharmaceutical Co. (Changchun, China) and the purity quotient was ≥99.5%.

A total of 48 rabbits were randomly assigned into 4 groups with the aid of a computer-generated table of random numbers; each group was restricted to 12 rabbits. In Group 1, Rg3 was injected into the tumor via the hepatic artery, in Group 2, lipiodol was injected, in Group 3, a cocktail of Rg3 mixed with lipiodol was injected, and in Group 4 saline was injected.

### Intervention technique and tissue preparation

The rabbits were anesthetized with ketamine (40 mg/kg) and xylazine (5 mg/kg) by intramuscular injection. Access to the right common femoral artery was obtained via surgical cutdown, where a 2.7/2.9 Fr microcatheter (Terumo, Tokyo, Japan) was introduced. Arteriography of the common hepatic artery was performed to demonstrate the hepatic arterial anatomy and the location, size and vascularity of the tumor. The catheter was selectively advanced into the tumor feeding artery in which, depending on the subject’s group, Rg3 (6.0 mg/kg), lipiodol (0.1 ml/kg) or a mixture of both was injected through the catheter until a reduction of blood flow to the tumor was observed. In the case of Group 4, 1 ml saline was injected through the catheter in each subject. The catheter was then removed and the common femoral artery was ligated with absorbable sutures.

Two weeks later, all rabbits were sacrificed with high-dose ketamine. The tumor tissue was carefully dissected and divided into two sections. One of the sections was placed in 4% paraformaldehyde and 0.1 mol/l phosphate-buffered saline at pH 7.0 and 4°C for 24 h and then embedded in paraffin, and the other was frozen in liquid nitrogen and stored at −80°C until further analysis. Paraffin sections (5 *μ*m thick) were mounted on poly-L-lysine-coated slides and stained with hematoxylin and eosin for observation under a microscope.

### Immunohistochemistry

To investigate the angiogenesis and cell apoptosis in the tumor, we detected the expression of CD31, VEGF and caspase-3. The sections were deparaffinized in xylene and immersed in graded ethanol and distilled water. Immunohistochemical staining was performed using the avidin-biotin peroxidase complex (ABC) method according to the manufacturer’s instructions (Dako, Carpinteria, CA, USA). The primary antibodies for CD31 (Abcam, Boston, MA, USA), VEGF (Abcam) and caspase-3 (Santa Cruz Biotechnology, Inc. Santa Cruz, CA, USA) were diluted to 1:500. The primary antibody was omitted as a negative control for the immunostaining. An image of each section was captured using a light microscope (Olympus, Tokyo, Japan) at ×400 magnification and the integrated optical density (IOD) of the positively stained tissue in each image was determined using Image Pro Plus software, version 6.0 (Media Cybernetics, Silver Spring, MD, USA).

### Cell culture

The human hepatocellular carcinoma cell line HepG2 was cultured in Dulbecco’s modifed Eagle’s medium (DMEM) routinely supplemented with 10% fetal bovine serum plus ampicillin and streptomycin, and incubated in 5% CO_2_ at 37°C.

### MTT assay

The MTT assay was performed to examine cell proliferation. Cells in the logarithmic phase were collected, and were seeded into 96-well plates, 5×10^3^ cells per well with 100 *μ*l medium, and the plates were incubated at 37°C in a humidified incubator with 5% CO_2_ for 24 h. The medium was removed and 100 *μ*l medium with different concentrations of Rg3 was added to each well. A control group and four Rg3 groups were created, the final concentrations of Rg3 were 0, 25, 50, 75 and 100 mg/l. Each group had six replicates. MTT reagent (20 *μ*l; Sigma, St. Louis, MO, USA) was added to each well at 12 h, 24 h, 36 h and 48 h. After culturing the cells for 4 h, the medium was removed and 150 *μ*l DMSO was added to each well and incubated for 15 min. The absorbance of the plates at 490 nm were read using a microplate reader (Model 680, Bio-Rad, Hercules, CA, USA). Experiments were performed in triplicate in three independent experiments.

### RNA isolation and semi-quantitative RT-PCR

Tumor tissues were rapidly excised and powdered in liquid nitrogen. Total RNA was extracted using TRIzol (Invitrogen, Carlsbad, CA, USA) according to the manufacturer’s instructions. For semi-quantitative RT-PCR, cDNA synthesis and PCR analysis were performed using the RevertAid™ First Strand cDNA Synthesis kit (Fermentas, Thermo Fisher Scientific Inc., Waltham, MA, USA). PCR was performed using an Eppendorf Mastercycler gradient thermal cycler (Eppendorf, Hamburg, Germany). The reaction cycle consisted of a hot start at 95°C for 10 min, then 30 cycles of denaturation at 95°C for 30 sec, annealing at 60°C for 30 sec and extension at 72°C for 30 sec. The primer sequences used for PCR were as follows: β-actin: 5′- C T T C CAG C C C T C C T T C C T - 3 ′ and 5′-GCCCGACTCGTCATACTCC-3′ (product size: 316 bp); caspase-3: 5′- CAATGGACTCTGGGAAAT-3′ and 5′-GCAAGCCTGAATAATGA-3′ (product size: 489 bp); Bax: 5′-CCAAGAAGCTGAGCGAGTG-3′ and 5′-TTCCAG ATGGTGAGTGAGG-3′ (product size: 400 bp); and Bcl-2: 5′-GTGGGATACTGGAGATGAAGA-3′ and 5′-GACGGT AGCGACGAG AGA-3′ (product size: 233 bp). Experiments were performed in triplicate in three independent experiments.

Following PCR amplification, 6 *μ*l PCR products plus 1 *μ*l loading buffer were run on 1.5% agarose gel containing 1 *μ*g/ml ethidium bromide. The PCR products were visualized and scanned using a Gel imaging system (Bio-Rad) and the intensity of each band present in each lane was determined using Quantity One software (Bio-Rad).

### Western blot analysis

To confirm the effect of each treatment on tumor cell apoptosis, the expression of the apoptosis-related genes, caspase-3, Bax and Bcl-2, were detected by semi-quantitative RT-PCR and the respective proteins were detected by western blot analysis. For protein extraction, tumor tissue was ground into powder in liquid nitrogen with a pre-cooled mortar and pestle. The powdered tissue was homogenized in 500 *μ*l lysis buffer (50 mmol/l Tris-HCl, pH 7.4, 1% NP-40, 0.25% sodium deoxycholate, 150 mmol/l NaCl, 1 mmol/l EDTA, 1 mmol/l phenylmethylsulfonyl fluoride, 1 mmol/l Na_3_VO_4_, and 1 mmol/l NaF) and incubated at 4°C for 30 min, followed by centrifuging at 10,000 × g at 4°C for 20 min. The lysates were collected and the protein concentration was determined using the BCA Protein Assay kit. Equal amounts of proteins were separated by SDS-PAGE and transferred to polyvinylidene difluoride membranes (GE Healthcare Biosciences). The membranes were incubated with primary antibodies against caspase-3 (1:1000; Santa Cruz Biotechnology), Bax (1:1000; Sigma), Bcl-2 (1:1000; Sigma) or VEGF (1:1000; Santa Cruz Biotechnology). Antibody binding was revealed by incubation with horse-radish peroxidase-conjugated secondary antibodies (Santa Cruz Biotechnology) and an ECL Plus immunoblotting detection system (GE Healthcare Biosciences, Fairfield, CT, USA). Signals were quantified using NIH ImageJ 1.63 software (National Institutes of Health, Bethesda, MD, USA).

### Statistical analysis

Data were analyzed and calculated with one-way ANOVA using SPSS version 11.0 software (SPSS, Inc., Chicago, IL, USA) and were expressed as the mean ± standard deviation. P<0.05 was considered to indicate a statistically significant result.

## Results

### Rg3 inhibits the proliferation and VEGF expression of hepatocellular carcinoma cells in vitro

**Figure 1.**
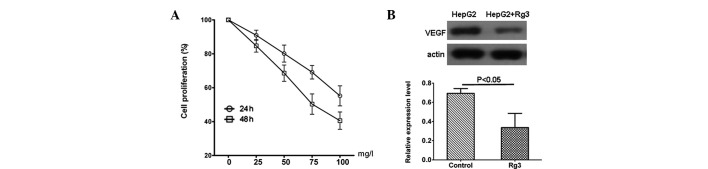
Effect of Rg3 on the growth of HepG2 human hepatocellular carcinoma cells. (A) The MTT assay showed that Rg3 inhibited the HepG2 cell proliferation; (B) western blotting showed that Rg3 downregulated VEGF expression in the HepG2 cells. Experiments were performed in triplicate in three independent experiments.

To determine whether Rg3 functions as a novel chemotherapeutic agent for human hepatocellular carcinoma, we first examined the effect of Rg3 on the proliferative activity of human hepatocellular carcinoma cells. As shown in Fig. 1A, the proliferation of human hepatocellular carcinoma cell line HepG2 was inhibited by Rg3 in a concentration-dependent manner *in vitro*. In addition, western blotting results indicated that Rg3 inhibited the VEGF expression of HepG2 cells significantly (P<0.05; Fig. 1B).

### TAE combined with Rg3 inhibits tumor growth in vivo

A total of 48 rabbits with VX2 liver tumors were included in this experiment. There was no significant difference in the mean volume of the tumor between each group prior to intervention. Following treatment, the tumors continued to increase in volume in each group. At 2 weeks, the mean tumor volume and growth rate were significantly lower in Groups 2 (TAE) and 3 (Rg3 and TAE) than in the control (P<0.05). In addition, the mean tumor growth rate in the rabbits treated with Rg3 and TAE was significantly lower than that in the TAE group (P<0.05). There was no significant difference in the mean tumor growth rate between Group 1 (Rg3) and the control (P>0.05), and notably, no adverse-effects were observed in this experiment (Table I).

### TAE combined with Rg3 inhibits expression of CD31 and VEGF in vivo

**Figure 2.**
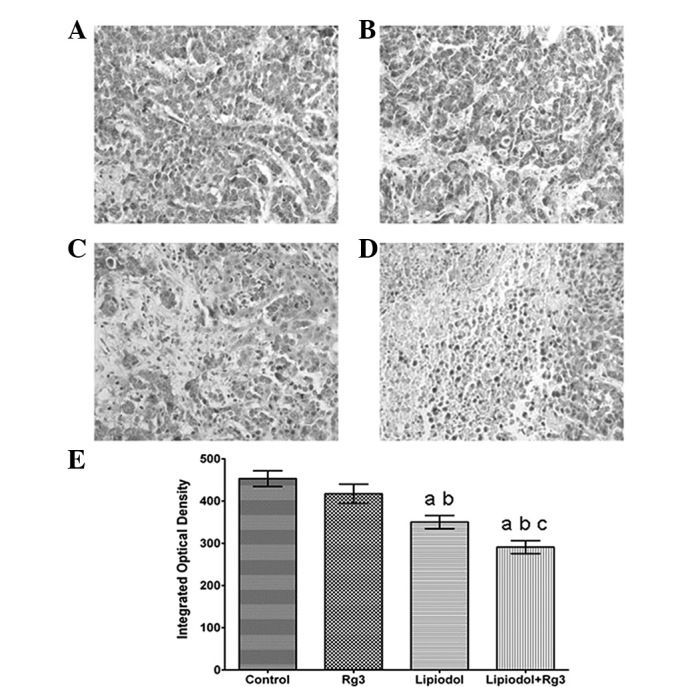
Immunohistochemical staining detected VEGF expression in tumor tissue. (A) Control; (B) Group 1 (Rg3); (C) Group 2 (lipiodol); (D) Group 3 (Rg3 and lipiodol); (E) integrated optical density of each group. ^a^P<0.05 vs. control; ^b^P<0.05 vs. Group 1 (Rg3); ^c^P<0.05 vs. Group 2 (lipiodol).

**Figure 3.**
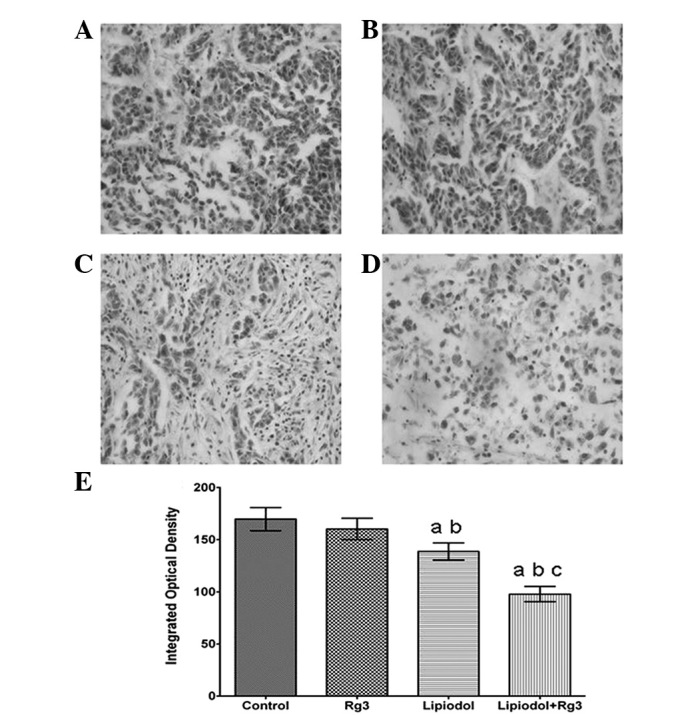
Immunohistochemical staining detected CD31 expression in tumor tissue. (A) Control; (B) Group 1 (Rg3); (C) Group 2 (lipiodol); (D) Group 3 (Rg3 and lipiodol); (E) integrated optical density of each group. ^a^P<0.05 vs. control; ^b^P<0.05 vs. Group 1 (Rg3); ^c^P<0.05 vs. Group 2 (lipiodol).

**Figure 4.**
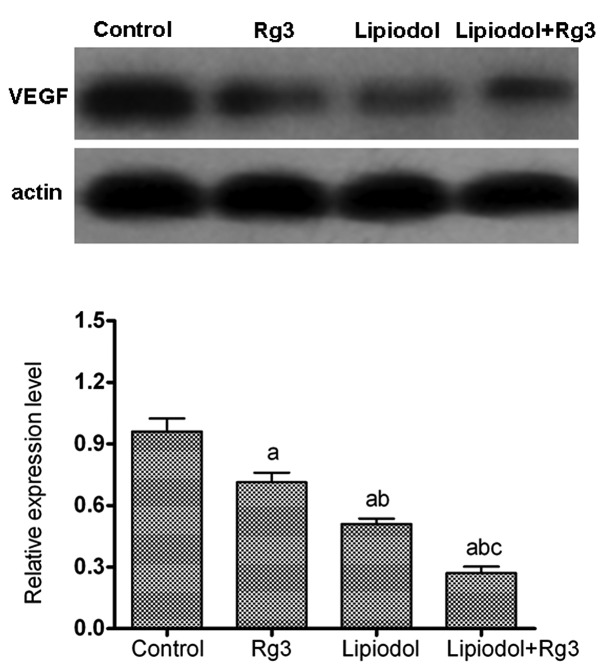
Western blotting detected the VEGF expression in tumor tissue. ^a^P<0.05 vs. control; ^b^P<0.05 vs. Group 1 (Rg3); ^c^P<0.05 vs. Group 2 (lipiodol). Experiments were performed in triplicate in three independent experiments.

Immunohistochemical analysis demonstrated that the tumor tissues were weakly stained for VEGF (Fig. 2) and CD31 (Fig. 3) antibody in Group 2 (TAE) and particularly weakly in Group 3 (Rg3 and TAE) compared with Group 1 (Rg3) and the control. Groups 2 (TAE) and 3 (Rg3 and TAE) each had a significantly lower integrated optical density (Fig. 2E and Fig. 3E) than the other groups. Group 1 (Rg3) also had a lower integrated optical density than the control group, however, there was no significant difference between the two groups. A western blot analysis of VEGF (Fig. 4) showed similar results to those of the immunohistochemical analysis. The mean relative protein expression of VEGF was 0.95±0.10, 0.71±0.07, 0.50±0.08 and 0.26±0.04 in the control group, Rg3 group, lipiodol group and lipiodol + Rg3 group, respectively (Fig. 4). The expression level of VEGF in the group treated with TAE and Rg3 was significantly decreased compared with that in the other groups (P<0.05; Fig. 4).

### TAE combined with Rg3 upregulates the expression of apoptosis-related genes in vivo

**Figure 5.**
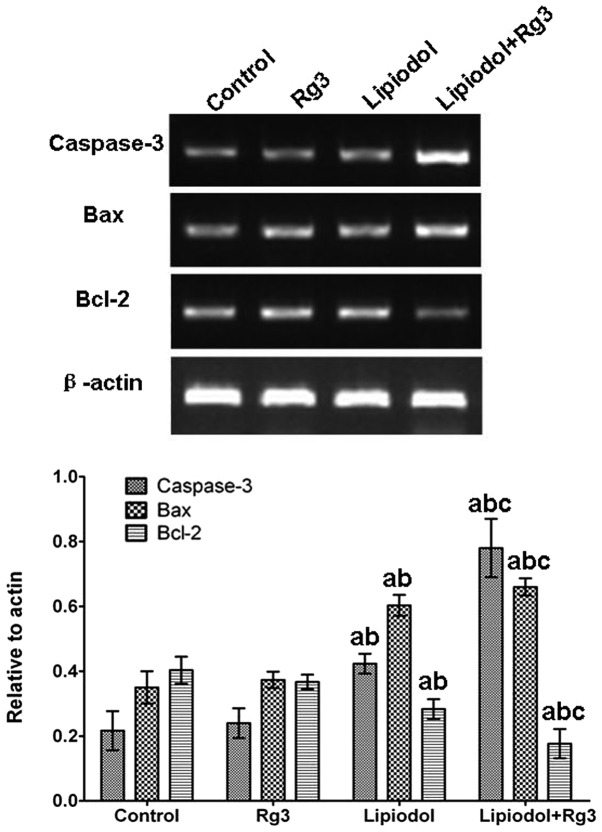
Semi-quantitative RT-PCR analysis of the caspase-3, Bax and Bcl-2 mRNA level in each group. ^a^P<0.05 vs. control; ^b^P<0.05 vs. Group 1 (Rg3); cP<0.05 vs. Group 2 (lipiodol). Experiments were performed in triplicate in three independent experiments.

**Figure 6.**
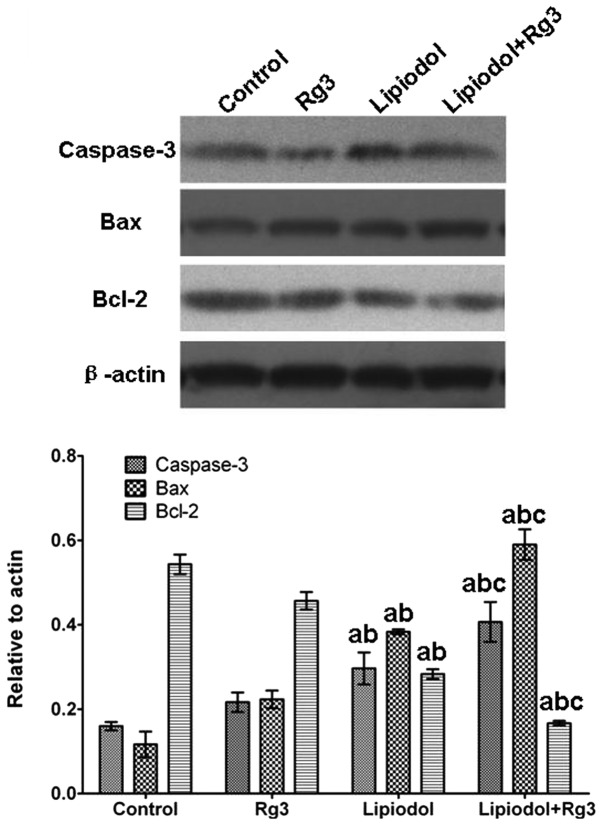
Western blot analysis of the caspase-3, Bax and Bcl-2 protein level in each group. ^a^P<0.05 vs. control; ^b^P<0.05 vs. Group 1 (Rg3); ^c^P<0.05 vs. Group 2 (lipiodol). Experiments were performed in triplicate in three independent experiments.

**Figure 7.**
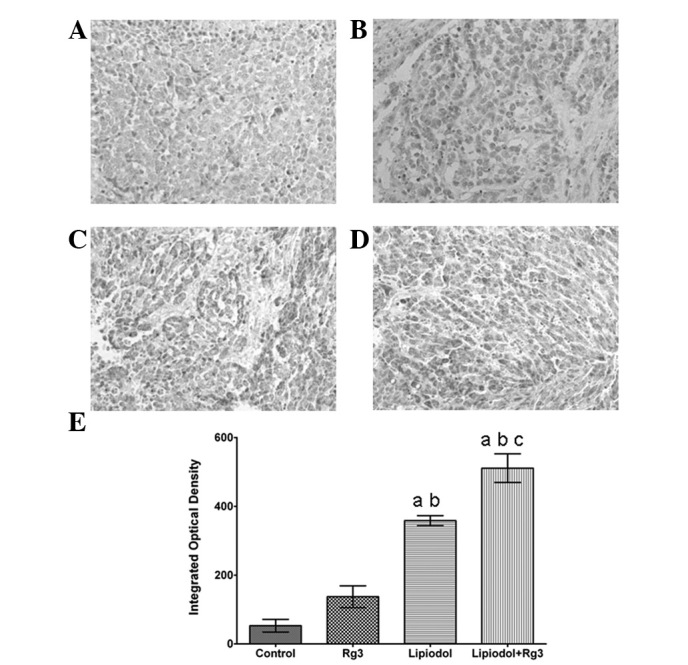
Immunohistochemical staining detected caspase-3 expression in each group. (A) Control; (B) Group 1 (Rg3); (C) Group 2 (lipiodol); (D) Group 3 (Rg3 and lipiodol); (E) integrated optical density of each group. ^a^P<0.05 vs. control; ^b^P<0.05 vs. Group 1 (Rg3); ^c^P<0.05 vs. Group 2 (lipiodol).

Caspase-3 and Bax expression levels in tumor tissues from Group 3 (Rg3 and TAE) were significantly increased compared with those from the other groups at the mRNA (Fig. 5) and protein (Fig. 6) levels. Group 2 (TAE) also had significantly increased expression levels compared with the control group, but the increase was inferior compared with that in Group 3 (Rg3 and TAE). No significant difference was observed between Group 1 (Rg3) and the control. With respect to Bcl-2, the expression level in Group 3 (Rg3 and TAE) was significantly lower than in the other groups. By contrast, the control group and Group 1 (Rg3) had significantly higher Bcl-2 levels and there was no significant difference in Bcl-2 level between Group 1 (Rg3) and the control. The immunohistochemical staining of caspase-3 also confirmed these results (Fig. 7).

## Discussion

The most prominent constituents of ginseng, a traditional Chinese medicinal herb, are ginsenosides. Thus far, over 40 types of ginsenosides have been isolated and have been demonstrated to affect the central nervous system and immune system, thereby producing anti-stress, anti-ageing, anti-fatigue, anti-hyperlipemic and anti-angiogenic effects (9–12). Of all ginsenosides isolated from ginseng, ginsenoside Rg3 has gained much attention for its antitumor properties (13). Previous studies have shown that Rg3 may inhibit cancer cell proliferation, inhibit metastasis, promote cancer cell apoptosis and enhance immunity (14–17). Kim *et al* demonstrated that chemotherapeutics combined with Rg3 may have enhanced therapeutic efficacy and reduced side-effects (18). At present, the oral administration of Rg3 is employed by physicians worldwide. However, the therapeutic effect of Rg3 when administered selectively via an artery has not been investigated.

Numerous studies in recent years have revealed that angiogenesis is essential for the growth of cancer in solid tumors (4,5). Angiogenesis plays an important role in tumor progression; it is responsible for accelerated cancer cell replication and may also promote tumor metastasis (4). For these reasons, the inhibition of angiogenesis in solid tumors is an attractive target for cancer therapy. Arterial embolization is an effective method for the treatment of liver tumors, but its long-term efficacy remains limited (3). It has been suggested that ischemia and hypoxia induced by TAE stimulates tumor angiogenic activities (5,19), which may partially explain the poor long-term efficacy and the frequent requirement for TAE to be repeated (20).

In the present study, TAE therapy was combined with selective arterial administration of Rg3 for the treatment of VX2 liver tumors in rabbits. Compared with either Rg3 or TAE monotherapy, the combination of the two modalities may achieve a greater therapeutic outcome. While TAE congests the microvessels feeding the tumor, Rg3 may inhibit angiogenic activities caused by tumor ischemia and hypoxia. In addition, the hepatic arterial administration of Rg3 combined with TAE increases the local concentration of Rg3, which may also facilitate therapeutic efficacy. The results of the present study were in agreement with this hypothesis and demonstrated that the hepatic arterial administration of Rg3 combined with TAE has a better therapeutic effect than either therapy alone.

Immunohistochemistry and western blot analysis demonstrated that VEGF and CD31 expression in Groups 2 (TAE) and 3 (Rg3 and TAE) was significantly lower than in the control group. Group 3 (Rg3 and TAE) displayed a lower VEGF and CD31 expression than Group 2 (TAE). These results indicate that Rg3 may inhibit angiogenesis following TAE. Previous studies have indicated that TAE enhances tumor hypoxia, which upregulates the expression of VEGF (1–3). Notably, in the present study, the VEGF expression was significantly downregulated in the group treated with TAE compared with the control group. This may be due to the TAE therapy causing the necrosis and shrinkage of tumors. The results of histological and morphological examination were also confirmed by the necrosis or apoptosis of tumor cells observed in the group treated with TAE. A previous study has shown that the rate of cancer cell proliferation and apoptosis is closely correlated with the progression of cancer (21), therefore, pro-apoptotic and anti-apoptotic genes were monitored in order to evaluate the antitumor and anti-angiogenic activity of Rg3 and TAE. The results of the present study showed that TAE alone and hepatic arterial administration of Rg3 in combination with TAE may downregulate the Bcl-2 expression and upregulate the caspase-3 and Bax expression significantly. Compared with TAE monotherapy, there was a significantly lower Bcl-2 expression and higher caspase-3 and Bax expression in Group 3 (Rg3 and TAE). These results suggest that selective hepatic arterial administration of Rg3 in combination with TAE may significantly inhibit tumor cell proliferation and promote tumor cell apoptosis through a caspase-dependent mechanism.

This study investigated the therapeutic efficacy of hepatic arterial administration of Rg3 combined with TAE for the treatment of VX2 liver cancer in rabbits. The results demonstrated that Rg3 combined with TAE may induce VX2 liver tumor cell apoptosis and inhibit angiogenesis, and showed that it is an effective and safe method for the treatment of VX2 liver tumors in rabbits. However, the application of Rg3 combined with TAE in the clinic requires further investigation.

## Figures and Tables

**Table I.** Pre- and post-treatment tumor volume and post-treatment tumor growth rate in each group at 2 weeks.

Group	Tumor volume (cm^3^) pre-treatment	Tumor volume (cm^3^) post-treatment	Tumor growth rate (%) post-treatment
Rg3	1.89±0.57	3.72±1.09	196.80±21
Lipiodol	2.04±0.98	2.98±1.29^a^	146.07±14^a^,^b^
Lipiodol+Rg3	2.07±0.85	2.54±1.07^a–c^	117.05±12^a–c^
Control	1.96±0.68	3.95±1.91	201.50±15

a P<0.05 vs. Group 4 (control);

b P<0.05 vs. Group 1 (Rg3);

c P<0.05 vs. Group 2 (lipiodol). Data are expressed as mean ± standard deviation.
